# Early data from project engage: a program to identify and transition medically hospitalized patients into addictions treatment

**DOI:** 10.1186/1940-0640-7-20

**Published:** 2012-09-25

**Authors:** Anna Pecoraro, Terry Horton, Edward Ewen, Julie Becher, Patricia A Wright, Basha Silverman, Patty McGraw, George E Woody

**Affiliations:** 1 Perelman School of Medicine, University of Pennsylvania, Philadelphia, PA, USA; 2 Clinical Trials Network, Delaware Valley Node, Wilmington, DE, USA; 3 Christiana Care Health System and Wilmington Hospital, Wilmington, DE, USA; 4 Delaware Physicians Care Incorporated, Wilmington, DE, USA; 5 Brandywine Counseling and Community Services, Wilmington, DE, USA; 6 Bryn Mawr Graduate School of Social Work and Social Research, Bryn Mawr, PA, USA

**Keywords:** Addiction, Drug, Alcohol, Hospital, Medical patients, Brief intervention, Facilitated referral to treatment, SBIRT, BI, Treatment initiation

## Abstract

**Background:**

Patients with untreated substance use disorders (SUDs) are at risk for frequent emergency department visits and repeated hospitalizations. Project Engage, a US pilot program at Wilmington Hospital in Delaware, was conducted to facilitate entry of these patients to SUD treatment after discharge. Patients identified as having hazardous or harmful alcohol consumption based on results of the Alcohol Use Disorders Identification Test-Primary Care (AUDIT-PC), administered to all patients at admission, received bedside assessment with motivational interviewing and facilitated referral to treatment by a patient engagement specialist (PES). This program evaluation provides descriptive information on self-reported rates of SUD treatment initiation of all patients and health-care utilization and costs for a subset of patients.

**Methods:**

Program-level data on treatment entry after discharge were examined retrospectively. Insurance claims data for two small cohorts who entered treatment after discharge (2009, n = 18, and 2010, n = 25) were reviewed over a six-month period in 2009 (three months pre- and post-Project Engage), or over a 12-month period in 2010 (six months pre- and post-Project Engage). These data provided descriptive information on health-care utilization and costs. (Data on those who participated in Project Engage but did not enter treatment were unavailable).

**Results:**

Between September 1, 2008, and December 30, 2010, 415 patients participated in Project Engage, and 180 (43%) were admitted for SUD treatment. For a small cohort who participated between June 1, 2009, and November 30, 2009 (n = 18), insurance claims demonstrated a 33% ($35,938) decrease in inpatient medical admissions, a 38% ($4,248) decrease in emergency department visits, a 42% ($1,579) increase in behavioral health/substance abuse (BH/SA) inpatient admissions, and a 33% ($847) increase in outpatient BH/SA admissions, for an overall decrease of $37,760. For a small cohort who participated between June 1, 2010, and November 30, 2010 (n = 25), claims demonstrated a 58% ($68,422) decrease in inpatient medical admissions; a 13% ($3,308) decrease in emergency department visits; a 32% ($18,119) decrease in BH/SA inpatient admissions, and a 32% ($963) increase in outpatient BH/SA admissions, for an overall decrease of $88,886.

**Conclusions:**

These findings demonstrate that a large percentage of patients entered SUD treatment after participating in Project Engage, a novel intervention with facilitated referral to treatment. Although the findings are limited by the retrospective nature of the data and the small sample sizes, they do suggest a potentially cost-effective addition to existing hospital services if replicated in prospective studies with larger samples and controls.

## Background

Alcohol and drug use are associated with a variety of medical conditions [1,2] and carry high global burdens of disease, injury, and cost [3,4]. Substance use is associated with inadequate ambulatory care utilization and poor health outcomes [5], and people with substance use are over-represented among frequent consumers of emergency department (ED) [6] and inpatient [7] medical services. Substance abuse is predictive of discharge against medical advice [8], and inpatients discharged with substance use disorder (SUD) diagnoses, particularly drug-related diagnoses, have higher rates of recurrent ED and medical inpatient service utilization [9]. This is not only associated with unnecessary human suffering but also generates disproportionately high health-care costs [10].

Hospital medical units are aggregators of people with SUDs, and hospitalization itself could serve as a “reachable moment” to intervene with these patients and engage them in appropriate SUD treatment after discharge [11]. In-hospital interventions to help patients enter SUD treatment might improve this situation, and such programs are likely to receive heightened attention since the Patient Protection and Affordable Care Act [12] will reduce Medicare payments to hospitals with excess readmissions beginning in October 2012.

In September 2008, leadership at Wilmington Hospital in the US state of Delaware collaborated with Brandywine Counseling and Community Services (BCCS), a major provider of SUD treatment in Delaware, to develop and implement Project Engage, a pilot program to identify medical and surgical inpatients with problematic substance use and to help them enter SUD treatment after discharge. Wilmington Hospital is a 241-bed general hospital owned and operated by Christiana Care Health System (CCHS), one of the largest health-care providers in the US mid-Atlantic region. Christiana Care Health System serves the state of Delaware and portions of seven New Jersey, Pennsylvania, and Maryland counties. In 2011, Wilmington Hospital recorded 52,178 ED visits and 13,778 medical and surgical admissions.

Project Engage has its theoretical basis in the literature on brief intervention (BI) to address excessive alcohol use among primary care outpatients [13]; BI for risky drinking and alcohol dependence among medical inpatients [14,15]; and screening, BI, and referral to treatment (SBIRT) for patients with moderate to high risk alcohol and/or drug use or dependence in diverse medical settings, including primary care, EDs, trauma centers, and inpatient and outpatient medical hospital services [16-18].

Studies reported in this literature have had promising outcomes. Patients in a large, federally funded SBIRT study conducted in six states reported decreases in illicit drug and heavy alcohol use subsequent to participation [16]. Studies of SBIRT in EDs have demonstrated decreased health-care costs and inpatient utilization [17] and increased rates of admissions to SUD treatment [19]. Randomized trials of BI for excessive alcohol use among primary care outpatients [13] have shown significant reductions in self-reported drinking. Data from screening and BI (SBI) for primary care outpatients with unhealthy nondependent alcohol use [13] led the US Joint Commission on Accreditation of Healthcare Organizations (JCAHO) to include performance measures for its use in hospitals [20].

Although these lines of research are significant, they have important gaps. For example, most published studies have applied BI to patients with unhealthy or risky drinking, alcohol abuse, and/or alcohol dependence. In reality, alcohol and drug problems are frequently comorbid, and patients with alcohol and drug problems—or primary drug problems—are also in need of care. Further, the majority of BI studies demonstrated efficacy in reducing alcohol use when alcohol-dependent individuals were excluded [21,22]; however, patients with alcohol dependence constitute the majority of medical inpatients with alcohol problems [23] and have a great need for SUD treatment. A literature search revealed a paucity of published studies of alcohol and drug BI or SBIRT conducted exclusively with hospital inpatients. Finally, hospitalized patients with SUDs often face multiple barriers to accessing treatment including homelessness, brief lengths of stay complicating discharge planning, ambivalence, and inadequate transfer resources [24]. These problems require an increased emphasis on referral to treatment. Since the chances of engaging patients in treatment decrease with the length of time between assessment and treatment admission [25], facilitated admission could be particularly important for this population.

### Description of the project engage pilot program

In many cases, SUDs directly or indirectly contribute to health problems leading to hospitalization. Patients with SUDs are often well known to hospital staff, but clinical teams typically have little training or experience in addressing SUDs. In fact, hospital personnel are often frustrated with these patients due to frequent rehospitalizations, noncompliance with recommendations to cut back or abstain, and resistance to entering and staying in SUD treatment. Project Engage, a modified version of BI and SBIRT, was designed to provide bedside assistance for the clinical team to address these problems. It consists of SUD identification by hospital staff based on clinical impressions but without a universal standardized screening process to identify alcohol and drug problems, followed by BI and facilitated referral to treatment (FRT). Although there are efforts to identify patients, this does not constitute “screening” because a universal, standardized approach to identification is not employed. Referral to treatment is enhanced by facilitation. The Project Engage pilot program described here was not designed as a research study, although self-report data on initiation of SUD treatment by Project Engage patients after discharge were collected, and insurance-claims data on two small cohorts of patients were examined retrospectively.

### Identification

Hospital clinical staff identified patients with possible alcohol and/or drug problems per usual procedures. Before Project Engage was initiated, brief trainings were provided to nursing staff on how to identify patients with problematic drug or alcohol use. The potential value of connecting them to treatment was emphasized, and an overview of the Project Engage program along with contact information for Project Engage staff was provided. In October 2009, the Alcohol Use Disorders Identification Test-Primary Care (AUDIT-PC) [26-28], a five-item self-report instrument to detect “hazardous and harmful alcohol consumption [29],” was initiated system-wide at CCHS to detect patients at risk for alcohol withdrawal and delirium tremens (DTs), and nursing staff administered it to all medical/surgical inpatients at admission.

Patients were identified for possible inclusion in Project Engage if they met any of the following criteria: clinical suspicion of alcohol and/or drug abuse or dependence; hospital admission likely related to alcohol and/or drug abuse or dependence; positive result on a drug test; AUDIT-PC ≥ 5 (as of October 2009); primary, secondary, or tertiary diagnosis related to substance use; or self-reported past or current alcohol and/or drug use. Patients under age 18 or with senility, dementia, or other disorders that interfered with the ability to provide informed consent to be seen by a non-CCHS provider were excluded from Project Engage. Nursing staff provided eligible patients with a choice to participate—or not participate—in Project Engage. Although Project Engage was not a research study, patients who chose to participate in it signed a “Choice Form” as part of an informed-consent process required in order to be seen by a non-CCHS provider. (The patient engagement specialists [PESs] were employed by BCCS.) Unfortunately, the number of patients who were identified and approached for participation, the number of interventions received by each patient, and the number of Project Engage patients who were unwilling to accept a referral were not recorded.

### Brief intervention

Patients who chose to participate in Project Engage received a BI from a PES hired specifically for the project. Project Engage specialists were in stable recovery from alcohol and/or drugs (at least two years without drug or alcohol use) and selected on the basis of emotional stability, experience in recovery, and interpersonal strengths. They received training in working in a health-care setting, co-occurring disorders, rapport building, basic interviewing techniques, assessment, motivational interviewing (MI), treatment referral, and ethics and were regularly supervised by licensed chemical-dependency professionals.

The BI occurred while patients were hospitalized and consisted of rapport building, a brief assessment, and one or two brief motivational interviewing (MI) sessions [30] to enhance patient motivation to attend SUD treatment and accept a facilitated referral. The purpose of the assessment was to determine if patients might benefit from SUD treatment and to identify possible barriers to transitioning them into it. The PESs used the Delaware Division of Substance Abuse and Mental Health (DSAMH) Co-Occurring Conditions Screening Instrument in conjunction with information gathered during MI sessions and the DSAMH/American Society of Addiction Medicine (ASAM) Crosswalk to match patient treatment needs to treatment programs according to ASAM’s Patient Placement Criteria-2nd Revision (ASAM PPC-2R [31]). If treatment slots in appropriate Delaware programs were not available, patients received facilitated referrals to programs in neighboring states.

### Facilitated referral to treatment

When patients were willing to consider SUD treatment, the PESs provided them with facilitated referrals as follows: They discussed potential treatment programs, and when patients agreed to consider a program, the PESs determined whether that program had an opening, whether it accepted the patient’s insurance or could admit him/her with other funding, and (if both these conditions were met) made an appointment for a time that was convenient to the patient. Patients who were in need of treatment and willing to accept a referral received a date and time for an appointment or inpatient admission rather than the name and phone number of a program. For programs that required the Addiction Severity Index [32], PESs administered it at bedside if patients were willing to complete it. The PESs also assessed potential barriers to treatment initiation such as homelessness, transportation difficulties, or lack of appropriate clothing. When necessary, patients were given bus or train tickets, driven to the treatment program, or picked up by the treatment program upon discharge. The PESs also contacted shelters for housing, acquired clothing for patients in need, and called patients within 48 hours after their scheduled admission or appointment to confirm that they attended. When patients reported having gone to treatment, PESs gave positive feedback and encouraged them to continue; when patients reported that they had not gone to treatment, PESs attempted to problem-solve any barriers and left the door open for future contact to facilitate admissions or appointments.

## Methods

The Project Engage pilot at Wilmington Hospital was not prospectively designed as a research study; however, program-level data on patients’ self-reported initiation of SUD treatment, as well as a description of health-care utilization before and after the intervention for two small cohorts of Project Engage patients who entered SUD treatment, were available from a single health plan and are presented here.

### Participants

Participants included all Project Engage patients seen between 9/1/2008 and 12/30/2010 (n = 415) as well as two smaller groups of patients who received the Project Engage intervention, initiated SUD treatment after discharge, and had uninterrupted insurance coverage and complete claims data three months before and three months after the intervention (2009 group) (n = 18) or six months before and six months after the intervention (2010 group) (n = 25).

Of the 415 patients seen between September 1, 2008, and December 30, 2010, 275 (65%) were male, and 135 (33%) were female (5 did not self-identify as either gender); 201 (48%) were white, 188 (45%) were black, and 26 (6%) self-identified as mixed race or other. The average age of patients was 46 years (SD, 11.8 years), and 183 (44%) were ≥50 years. Regarding their primary substance of choice (some were multiple), 240 (58%) reported alcohol, 90 (22%) reported crack or powder cocaine, 64 (15%) reported heroin, 17 (4%) reported marijuana, 11 (3%) reported an opioid other than heroin, 5 (0.01%) reported benzodiazepines, and 4 (0.01%) reported methamphetamines.

The two smaller cohorts consisted of all patients insured by Delaware Physicians Care Incorporated (DPCI) who had uninterrupted coverage and complete claims data. The 2009 cohort participated in Project Engage between June 1, 2009, and November 30, 2009, and consisted of nine men and nine women. The average age was 43 years (SD, 10 years). The 2010 cohort participated in Project Engage between June 1, 2010, and November 30, 2010, and consisted of 12 men and 13 women. The average age was 40 years (SD, 12 years). Unfortunately, the small number of patients meeting inclusion criteria (uninterrupted coverage and complete claims data) did not allow for random selection.

### Data analytic strategy

Brandywine Counseling and Community Services furnished program-level data on the number of patients who participated in Project Engage between September 1, 2008, and December 30, 2010, and on self-reported SUD treatment initiation after discharge. Delaware Physicians Care Incorporated provided claims data for two smaller cohorts. Christiana Care Health System’s Institutional Review Board approved queries to BCCS’s Project Engage records to determine rates of treatment initiation and the use of data from DPCI’s reports for a poster presentation [33] and this article. Unfortunately, the DPCI datasets from which the reports were generated were not available to the authors, so detailed health economic analyses were not possible.

## Results

### Program-level data: Participant admissions to SUD treatment

Between September 1, 2008, and December 30, 2010, 415 patients participated in Project Engage. (The number of patients identified and approached for participation was not recorded.) Of these patients, 180 (43%) were admitted to an inpatient treatment program and/or attended one or more session(s) at an outpatient program. Of these patients, 16 (8%) were admitted to inpatient detoxification; 53 (29%) were admitted to residential treatment; 103 (57%) were admitted to outpatient treatment; and 8 (4%) were admitted to transitional housing and treatment (Table 1).

**Table 1:** Admissions to substance abuse treatment for project engage patients seen between September 1, 2008, and December 30, 2010 (N = 415)

Admitted to a Substance Abuse Treatment Program	180 (43%)
- Inpatient Detoxification	16/180 (8%)
- Residential Treatment	53/180 (29%)
- Outpatient	103/180 (57%)
- Transitional Housing and Outpatient	8/180 (4%)

### Cohort-level data: Health-care utilization and costs before and after participation in project engage

Delaware Physicians Care Incorporated provided health-care utilization and costs for inpatient medical admissions, ED visits, and inpatient and outpatient behavioral health/substance abuse (BH/SA) admissions before and after the 2009 and 2010 subgroups received the Project Engage intervention (DPCI was not able to differentiate between BH and SA treatment in reported outcomes). The hospitalization during which patients received the Project Engage intervention was not included in these costs, but SUD treatment costs after hospitalization were included.

Of the 18 patients in 2009 subgroup who initiated SUD treatment after discharge, five had at least one BH/SA outpatient visit subsequent to the Project Engage intervention, and six had at least one inpatient BH/SA admission. There was a 33% ($35,938) decrease in inpatient medical admissions in this subgroup, a 38% ($4,248) decrease in ED visits, a 42% ($1,579) increase in BH/SA inpatient admissions, and a 33% ($847) increase in outpatient BH/SA admissions, for an overall cost decrease of $37,760 (Table 2).

**Table 2:** Health care utilization among patients in the 2009 and 2010 project engage subgroups

**Subgroup (N = 18)**			
	**Pre-Intervention (n)**	**Post-Intervention (n)**	**Difference**
Inpatient Medical Admissions	12	8	33% decrease ($35,938)
Emergency Department Visits	54	33	38% decrease ($4,248)
Inpatient Behavioral Health/Substance Abuse Admissions	7	10	42% increase ($1,579)
Outpatient Behavioral Health/Substance Abuse Admissions	12	16	33% increase ($847)
**2010 Subgroup (N = 25)**			
	**Pre-Intervention (n)**	**Post-Intervention (n)**	**Difference**
Inpatient Medical Admissions	17	7	58% decrease ($68,422)
Emergency Room Visits	133	116	12.7% decrease ($3,308)
Inpatient Behavioral Health/Substance Abuse Admissions	28	19	32% decrease ($18,119)
Outpatient Behavioral Health/Substance Abuse Admissions	25	33	32% increase ($963)

Of the 25 patients in the 2010 subgroup who initiated SUD treatment after discharge, 13 had at least one BH/SA outpatient visit subsequent to the Project Engage intervention, and 9 had at least one inpatient BH/SA admission. a 58% ($68,422) decrease in inpatient medical admissions; a 13% ($3,308) decrease in emergency department visits; a 32% ($18,119) decrease in BH/SA inpatient admissions, and a 32% ($963) increase in outpatient BH/SA admissions, for an overall decrease of $88,886 (Table 2).

## Discussion

Although this pilot program was not designed as a research study, retrospective evaluation of the data yielded useful descriptive information. Project Engage involved collaboration between a large hospital system, an SUD treatment provider, and a health plan and demonstrated that such collaboration is possible in a clinical setting. It also demonstrated that cost data (although limited) can be obtained outside the context of a formal research study. Importantly, FRT (a major component of Project Engage) is an innovative approach that warrants further study to assess its impact on treatment enrollment. The use of PESs rather than graduate students or licensed clinicians differs from approaches common in the existing BI and SBIRT literature. The success of Project Engage suggests interventions delivered by such individuals are accepted by patients and could be used in these and other settings.

The finding that a relatively large proportion (43%) of Project Engage patients entered SUD treatment after discharge is promising. Krupski et al. [19] examined admissions to treatment subsequent to BI (MI without referral to treatment) in ED patients who screened positive for alcohol and/or other drug problems and found that 34% of those who received the intervention were admitted to SUD treatment within 12 months compared with 23% of those who did not receive it. Saitz et al. [14] studied a BI (single MI session without referral to treatment) for medical inpatients with risky drinking or alcohol dependence and found that, among alcohol-dependent patients, 49% of the MI group and 44% of the control group attended alcohol treatment within three months; between-group differences were not significant. Our results are comparable but included patients who may have had alcohol *and/or* drug abuse and dependence and were collected within 48 hours of patients’ scheduled admissions or appointments. It is possible that these numbers could have changed three or 12 months after the intervention.

The preliminary findings concerning apparent differences in health-care costs before and after the intervention in the two smaller patient cohorts are also encouraging, as they reflect less medical and more BH/SA treatment utilization in the post-hospitalization period; however, they cannot definitively be considered cost savings due to a number of limitations. These include the absence of formal substance-use diagnoses; the lack of a control condition; the retrospective nature of data collection; the lack of data on the number of patients approached who declined to participate in Project Engage; the lack of data on the number of patients for whom a referral to treatment was not considered necessary; and differences in the specific characteristics of facilitated referrals. Because of these limitations, no conclusions can be drawn about causation. Also, the data on health-care costs were based on previously completed reports, the datasets for which were not released to the authors; thus further analyses were not possible.

Based on available findings, one can only conclude that a relatively large number (43%) of patients who received the Project Engage intervention entered SUD treatment, and differences in overall health-care costs were observed after participation. It is possible that these outcomes were affected by selection bias in that those patients who were most likely to participate in Project Engage and enter SUD treatment were also less likely to utilize medical health-care services. However, according to the literature, hospitalized patients like those who participated in Project Engage have not accessed substance abuse treatment by the usual referral processes. From the authors’ perspective, it is possible that engaging these patients in SUD treatment reduced their health-care utilization and costs by addressing their SUDs; however, this is impossible to prove due the limitations of these data. From the perspective of the payer, creating a portal for these patients to enter addiction treatment makes sense as a potential way to reduce health-care costs.

## Conclusions

Despite limitations, these results provide useful pilot data to justify prospective, controlled studies of similar interventions including FRT for medically hospitalized patients. Key next steps for Project Engage include refining the model to incorporate lessons learned; identifying potential sources of support; and examining potential pre-/post-participation differences in health-care costs with appropriate economic analyses. A standardized approach to screening may help hospital clinical staff to identify more patients in the future. It would also be useful to examine the role of FRT in greater detail. Randomized controlled trials comparing an intervention with FRT to an intervention without FRT are necessary. To increase the accuracy of endpoint measurement, it would be helpful to collect outcome data at six-months post-discharge that includes confirmation of attendance at treatment programs, self-reported substance use, urinalysis, and breath testing. Finally, this study looked at admissions to SUD treatment, not retention. It is well known that many patients admitted to addiction treatment do not remain in treatment [34]. Subsequent studies should investigate both admissions and retention. If retention is problematic, an adaptive continuing care component [35] could be added to future iterations of the Project Engage intervention. Due to these favorable initial findings described here and anonymous financial support, Project Engage was retained at Wilmington Hospital and initiated at Christiana Hospital in the fall of 2011. A prospective study of the intervention is underway.

## Competing interests

The authors declare that they have no competing interests.

## Authors’ contributions

AP coordinated inter-institutional collaboration for this program evaluation, reviewed the literature, drafted and edited the manuscript, guided queries on specific data points by BCCS and DPCI where possible, and prepared the manuscript for submission. TH developed the idea for Project Engage, collaborated with community partners, implemented the program at Wilmington Hospital, reviewed the literature, and reviewed and edited paper. EE reviewed and edited the manuscript. JB reviewed and edited the manuscript and helped to identify endpoints for future studies. PAW coordinated DPCI’s collaboration with Wilmington Hospital, provided reports on data analyses that were incorporated into the manuscript, and reviewed and edited the manuscript. BS led the BCCS team, trained and supervised the PESs, managed program-level data, participated in meetings between CCHS and BCCS staff, and reviewed and edited the manuscript. PM wrote the IRB application and reviewed and edited the manuscript. GW drafted and edited the manuscript. All authors read and approved the final manuscript.

## Financial support

This research was supported by a Christiana Care Community Services and Education Grant (Project Engage), NIDA grant U10 DA-13043 (AP), and NIDA U10 DA-13043 and KO5 DA-17009 (GW).
